# HISTÓRIA, CIÊNCIAS, SAÚDE – MANGUINHOS IN 2023: A YEAR OF CHANGE

**DOI:** 10.1590/S0104-59702024000100035en

**Published:** 2024-07-26

**Authors:** Roberta Cardoso Cerqueira, Marcos Cueto

**Affiliations:** i Executive editor, Casa de Oswaldo Cruz/Fiocruz. Rio de Janeiro – RJ – Brazil; ii Science Editor, Researcher, Casa de Oswaldo Cruz/Fiocruz. Rio de Janeiro – RJ – Brazil

As we have done before (for example, Cueto, 2023), once again we outline the main activities undertaken by our team in the previous year. The whole year was devoted to adapting to the new continuous publication format. This means that all the texts are now published in a single volume rather than in separate issues every quarter, as had been the case until 2022. The first year of this format was a learning experience that took a great deal of time and energy and involved some significant adjustments to our in-house editorial routines.

By the end of 2023, we had published 69 texts (counting all the sections except the letters from the editor), 18 of which in two languages. If we look at the institutional affiliation of the lead authors of the articles published in 2023, we can see that the majority were from Brazil, but there were also some important contributions by authors from other countries, notably Portugal and Spain, as well as, to a lesser extent, other countries in Latin America, the United States, France, the United Kingdom, China, and Singapore.

The continued internationalization of *História, Ciências, Saúde – Manguinhos* can be seen in the number of submissions received from authors from different countries, led by Portugal, Spain, and Argentina, even if the majority of the manuscripts still came from Brazil. This internationalization spread to the peer review process too, insofar as most of the manuscripts were reviewed by at least one non-Brazilian scholar with specialized knowledge in the subject matter. These data are important indicators of the increasingly international nature of the journal and a direct result of the commitment made since 2006 to have at least some of the articles translated into English.

We endeavor to publish articles of relevance to the areas within the scope of the journal. Among these, we would like to highlight one of the articles published in 2023, by Walter Francisco Figueiredo Lowande, from the Federal University of Alfenas, Brazil, entitled “Anthropocene, human sciences, and historiography.” In the text, Lowande examines the potential of new studies from the humanities to expand the explanatory horizons of this new planetary regime. Alongside our articles, we have an important Reviews section, edited by Ana Carolina Vimieiro-Gomes, from the Federal University of Minas Gerais, who oversaw the publication in 2023 of 19 reviews of books from Brazil, other Latin American countries, and other parts of the world. These reviews are fundamental for helping foster the communication of research and galvanize debate among specialists from Brazil and the region.

We would like to take this opportunity to invite researchers to send in book reviews containing critical analyses of volumes published in the last two years. Following the journal’s guidelines, these analyses should draw links between the central arguments presented in the book and contemporary debates, assessing how the book contributes to the development of the history of science and health and studies of historical heritage (Cueto, Vimieiro-Gomes, 2022).

In 2023, we published two supplements. The first, “Covid-19 in Latin America: conflicts, resistance, and inequalities,” had four guest editors: Claudia Agostoni, from the National Autonomous University of Mexico, Karina Ramacciotti, from the National University of Quilmes, and Carlos Henrique Paiva and Marcos Cueto, both from Casa de Oswaldo Cruz/Fiocruz. The articles analyzed the deficiencies in the official responses to the covid-19 pandemic, including debates about social inequality and the resilience of health workers. One of the texts in the supplement is an interview given by Deisy Ventura, a professor at the University of São Paulo’s Faculty of Public Health, in which she analyzes the official policy pursued in Brazil of systematically disseminating the virus. She argues that the pandemic should be addressed as a matter of memory, truth, and justice.

“Heritage and territory in Manguinhos” was the second supplement. Edited by Renato da Gama-Rosa Costa and Inês El-Jaick Andrade, researchers from the Department of Historical Heritage at Casa de Oswaldo Cruz, the supplement analyzed changes to the main Fiocruz campus. The result was an “interdisciplinary reflection on the relationship between territory and local identity, including tangible and intangible aspects, especially related to health and cultural heritage” (Costa, Andrade, 2023).

Some of the work undertaken by the *História, Ciências, Saúde – Manguinhos* team also deserves highlighting. This included a hybrid meeting held in April between the journal’s in-house editors and proofreaders and its outsourced proofreaders and translators, which included a presentation of: the changes made at the journal between 2019 and 2022; the new standards introduced ensure the journal complies with the premises of open science; and establish some updates of core documents used in-house, including the proofreading handbook, the translation standards, and the citation and reference guidelines. Another important step was the publication of updated author guidelines to bring them into line with the major US database PubMed Central, maintained by the National Library of Medicine. A third initiative of note was Mônica Auler’s participation in the meeting of the Brazilian Association of Scientific Editors, in November 2023, in Foz do Iguaçu, where she was able to network with editors and teams from scholarly journals around the country. Speakers at the event drew attention to opportunities artificial intelligence brings to scholarly communication and the challenges to mantain a journal in prestigious indexes and search engines. One other important step was the process of depositing the files for the articles published in 2021 and 2022 in the Fiocruz institutional repository, Arca, to ensure the preservation of the journal since it ceased to be published in hard-copy format (https://www.arca.fiocruz.br/handle/icict/54117).

Another important activity was the team’s participation in forums of scientific editors held in 2023. For example, *História, Ciências, Saúde – Manguinhos* engaged actively in meetings of the Forum of Editors of History Journals organized by the National Association of History. The forum participants discussed and drafted a document proposing criteria for the evaluation of periodicals in the area for the 2021-2024 Qualis Periódicos evaluation period. Qualis is the Brazilian system for evaluating scholarly journals, which is maintained by the government agency Capes. Throughout 2023, the team also took part in meetings held by the Fiocruz Editors’ Forum, which encompasses all the journals published by the institution as well as the Fiocruz publishing house, Editora Fiocruz. The meetings included discussions on how to ensure that editorial practices are aligned with the Fiocruz institutional mission.

In 2023 we continued to publicize our journal online and on social media. Our national blog now has more than 10,500 followers, and our international blog, which publishes news in English and Spanish, has some 5,300 followers. We also publish posts in English, Spanish, and Portuguese on events and news pertaining to the scope of the journal on Facebook. Parallel to this, we maintain a trilingual account on X (formerly Twitter), which now has 4,444 followers and has received over 800 retweets. Our experience indicates that the scholars who publish with *História, Ciências, Saúde – Manguinhos* can make a big difference in divulging the research published in our journal. For this reason, we would like to ask all our authors to include posts on their texts in these different media.

The adoption of a continuous publication model meant we had to adapt the way the journal was divulged, because instead of five issues a year, we started publishing a single volume, which we update as new texts become ready. Accordingly, we now divulge new articles as soon as they are published online. We also took advantage of some commemorative events to bring readers’ attention to articles published previously in *História, Ciências, Saúde – Manguinhos*. For example, to mark the 50th anniversary of Brazil’s National Vaccination Plan and the launch, by the Ministry of Health, of the National Vaccination Movement, we showcased articles on the history of vaccination in Brazil and Latin America. Likewise, World Food Day (October 16th) attracted a lot of visitors to the thematic dossier on the history of food published in 2021.

Throughout 2023, we consistently published three to four times per week on our social media, mostly with posts on new articles or events of interest in the history of science and heritage studies. One of the posts that had particular repercussions was an interview with Maria Paula Meneses, a researcher at the University of Coimbra’s Centre for Social Studies, in Portugal, who came to Rio de Janeiro to take part in the 8th Congress of the Network for Science and Technology Popularization in Latin America, held from July 10th to 16th, 2023, and organized by Museu da Vida (Museum of Life, Casa de Oswaldo Cruz/Fiocruz). We should add that three members of our editorial team – Marina Leme, Vivian Mannheimer, and Roberta C. Cerqueira – also attended this Congress, where they gave a presentation on *História, Ciências, Saúde – Manguinhos*’s blogs and social media to mark ten years since their inception.

We retain our commitment to publishing according to the precepts of open science, which implies making changes to the editorial process, such as the acceptance of manuscripts previously published on preprint servers. Unfortunately, most historians, journal editors included, are of the mind that preprints divulge unfinished research, challenge the unpublished status of journal articles, compromise the impartiality of the review process, and disclose research findings in a pace that is still foreign to the discipline. We would argue that editors should value and encourage the preprint format: not only does it shorten the time between the research per se and the communication of its findings to the public, but preprints have the potential to stimulate debate. They can also strengthen responsible and well documented reviewing and constitute a record of the initial formulation, data selection, and maturing of the ideas for scholarly research.

Another novelty introduced in 2023 in the way *História, Ciências, Saúde – Manguinhos* publishes its texts concerns articles with two or more authors. Information must now be provided in our articles on the contributions made by each author, in line with international criteria, covering points such as conceptualization, data collection, interpretation, and writing. This change is designed to ensure the ethical integrity of the text and make sure it is the outcome of a real partnership and not a case of supervision (as is sometimes unfortunately the case). Nonetheless, we are aware that history journals still need to make progress in their compliance with the principles of open science and respond to some complex challenges, such as promoting open peer reviews and fostering diversity and equity in the articles approved.

One challenge that faces *História, Ciências, Saúde – Manguinhos* in 2024 is the task of completing the migration to a new platform, the Open Journal Systems (OJS). This system enables the management of article submission, editorial process, online publication of the articles. As using the OJS allows the entire process to be conducted digitally, our printing costs have been reduced and we can continue to process articles submitted for publication free of charge.

We would like to end by noting that the progress made by the journal has only been possible thanks to our editorial team: Mônica Auler, Camilo Papi, Mônica Cruz Caminha, Vinícius Renaud, Marciel Mendonça, Miriam Junghans, Marina Lemle, Vivian Mannheimer, and Fernando Vasconcelos. We are extremely grateful to our authors, reviewers, section editors, and editorial board members, whose commitment has been crucial for our journal to gain such great visibility and prestige not only in Brazil but also elsewhere in Latin America and in other parts of the world. We extend our special thanks to Marcos José de Araújo Pinheiro, director of Casa de Oswaldo Cruz, Fiocruz, where we work, for the invaluable support given to the journal.
