# Supplementary material for: HISTÓRIA, CIÊNCIAS, SAÚDE – MANGUINHOS IN 2023: A YEAR OF CHANGE
Source: Hist Cienc Saude Manguinhos. 2024 Jul 26;31:e2024035. doi: 10.1590/S0104-59702024000100035en (PMC11277584; doi:10.1590/S0104-59702024000100035en)
Supplement: Translation

# Translation: HISTÓRIA, CIÊNCIAS, SAÚDE – MANGUINHOS EM 2023: UM ANO DE MUDANÇAS

**DOI:** 10.1590/S0104-59702024000100035

**Authors:** Roberta Cardoso Cerqueira, Marcos Cueto

**Affiliations:** i Editora executiva, Casa de Oswaldo Cruz/Fiocruz. Rio de Janeiro – RJ – Brasil; ii Editor científico, pesquisador, Casa de Oswaldo Cruz/Fiocruz. Rio de Janeiro – RJ – Brasil

Como fizemos em outros anos (por exemplo, Cueto, 2023), novamente informamos as principais atividades da revista no ano anterior. O período foi dedicado à mudança na publicação dos originais de *História, Ciências, Saúde – Manguinhos* para o formato de fluxo contínuo. Nesse modelo, os textos ficam reunidos em uma única edição, em vez de separados em números trimestrais, como fizemos até 2022. O primeiro ano desse formato foi um período de aprendizagem, que demandou tempo e aperfeiçoamento das rotinas editoriais internas do periódico.

Até o final de 2023, a revista publicou 69 textos (incluindo todas as seções, exceto as cartas do editor), sendo 18 bilíngues. Em relação à filiação institucional do primeiro autor dos artigos publicados em 2023, observamos o predomínio da autoria brasileira, mas tivemos contribuições importantes de outros países, especialmente Portugal e Espanha, e em menor medida de outros países latino-americanos e de EUA, França, Reino Unido, China e Cingapura.

Indicativo de que a revista seguiu seu processo de internacionalização foi o recebimento de originais de diversos países, com maior presença de autores provenientes de Portugal, Espanha e Argentina. Os números apontam, no entanto, que mais da metade dos originais foram do Brasil. Na maior parte dos textos avaliados, pelo menos um dos pareceristas era pesquisador estrangeiro conhecedor do assunto. Os dados apresentados são importantes para corroborar o processo de internacionalização e o esforço que *História, Ciências, Saúde – Manguinhos* tem feito, desde 2006, no sentido de traduzir para o inglês alguns de seus textos.

Buscamos publicar artigos de interesse para as pesquisas desenvolvidas no escopo da revista, e gostaríamos de destacar um dos artigos publicados em 2023, de autoria de Walter Francisco Figueiredo Lowande, da Universidade Federal de Alfenas (MG), intitulado “Antropoceno, ciências humanas e historiografia”. No texto, Lowande examinou o potencial dos novos estudos das ciências humanas para a ampliação dos horizontes explicativos desse novo regime planetário. Por outro lado, graças ao trabalho da nossa editora da seção Resenhas, Ana Carolina Vimieiro-Gomes, da Universidade Federal de Minas Gerais, publicamos 19 resenhas de livros brasileiros, latino-americanos e de outras partes do mundo, um número considerável que permitirá divulgar pesquisas e estimular o debate entre especialistas do país e da região.

Aproveitamos para convidar os pesquisadores e pesquisadoras a enviar resenhas, seguindo as instruções da revista, com análises críticas de obras publicadas nos últimos dois anos que relacionem a argumentação central do trabalho com os debates contemporâneos, ponderando a contribuição do livro para o desenvolvimento do campo de história das ciências e da saúde e os estudos de patrimônio histórico (Cueto, Vimieiro-Gomes, 2022).

Em 2023, publicamos dois suplementos. O primeiro, “Covid-19 en América Latina: conflictos, resistencias y desigualdades”, teve como editores convidados Claudia Agostoni, da Universidad Nacional Autónoma de México, Karina Ramacciotti, da Universidad de Quilmes, Carlos Henrique Paiva e Marcos Cueto, ambos da Casa de Oswaldo Cruz/Fiocruz. Os artigos analisaram as respostas oficiais insuficientes à pandemia de covid-19, com debates sobre a desigualdade social e a resiliência dos trabalhadores de saúde. Um dos textos do suplemento é a entrevista concedida por Deisy Ventura, professora da Faculdade de Saúde Pública da Universidade de São Paulo, que analisou a política oficial sistemática no Brasil, de disseminação do vírus. Segundo ela, a pandemia deveria ser tratada como uma questão de memória, verdade e justiça.

“Patrimônio e território em Manguinhos” foi o segundo suplemento. Organizado por Renato da Gama-Rosa Costa e Inês El-Jaick Andrade, pesquisadores do Departamento de Patrimônio Histórico da Casa de Oswaldo Cruz, o suplemento analisou as mudanças no *campus* principal da Fiocruz, estabelecendo uma “reflexão interdisciplinar sobre a relação entre território e identidade local, incluindo seus aspectos tangíveis e intangíveis, em especial na relação com a saúde e o patrimônio cultural” (Costa, Andrade, 2023).

Algumas atividades da equipe da revista também merecem destaque. Primeiro, a reunião híbrida dos revisores internos com revisores e tradutores externos realizada em abril, na qual foram apresentadas as inovações de *História, Ciências, Saúde – Manguinhos* no período de 2019 a 2022; as novas normas para adequar a revista à ciência aberta, e atualizações em documentos essenciais ao trabalho interno como nosso manual de revisão e as normas para tradução e elaboração de referências. Em segundo lugar, a atualização das instruções aos autores para cumprir exigências da importante base de dados norte-americana PubMed Central, mantida pela National Library of Medicine. Em terceiro, a participação de Mônica Auler no encontro da Associação Brasileira de Editores Científicos, chamado ABEC Meeting, em novembro de 2023, em Foz do Iguaçu, no Paraná, com objetivo de fazer contatos com editores e equipes de periódicos científicos de diferentes estados do Brasil. Palestras do evento focalizaram as oportunidades do uso de inteligência artificial na comunicação científica e os desafios para permanência de periódicos em indexadores de prestígio. Em quarto lugar, com a preocupação da preservação eletrônica da revista, os primeiros arquivos – artigos publicados em 2021 e 2022 – foram depositados no Arca, repositório institucional da Fiocruz (https://www.arca.fiocruz.br/handle/icict/54117).

Outra atividade importante desenvolvida pela equipe da revista foi a participação em fóruns de editores científicos que ocorreram em 2023. *História, Ciências, Saúde – Manguinhos* participou das reuniões promovidas pelo Fórum de Editores de História organizado pela Associação Nacional de História. Os integrantes desse fórum discutiram e elaboraram um documento sugerindo critérios de avaliação das revistas científicas da área para o Qualis Periódicos do quadriênio 2021-2024. O Qualis é o sistema brasileiro de avaliação das revistas científicas mantido pela Coordenação de Aperfeiçoamento de Pessoal de Nível Superior (Capes). Ao longo de 2023, a equipe também participou de reuniões promovidas pelo Fórum de Editores da Fiocruz, que reúne as revistas científicas editadas pela instituição mais a Editora Fiocruz, nos quais foram discutidas as práticas editoriais alinhadas à missão institucional.

Continuamos com a divulgação *online* da revista nas mídias sociais. O *blog* nacional conta com mais de 10.500 seguidores, e o *blog* internacional, que publica notícias em inglês e espanhol, atualmente tem 5.300 seguidores. Da mesma forma, postamos matérias em português, espanhol e inglês sobre eventos e notícias pertinentes ao escopo da revista no Facebook. Além disso, ainda temos uma ativa conta trilíngue no Twitter (agora X) que atualmente conta com 4.444 seguidores e já teve mais de 800 *retweets*. Nossa experiência indica que a divulgação dos próprios autores e autoras é fundamental para aumentar a visibilidade das pesquisas, por isso, gostaríamos de pedir aos autores e às autoras para divulgar seus textos nesses meios de comunicação.

O fluxo contínuo exigiu adaptações também na forma de divulgação que a revista adotou até 2022, já que deixamos de ter um volume com cinco números a cada ano. Com esse novo formato de publicação, passamos a divulgar os artigos à medida que eram publicados *online*. A passagem de efemérides foi atividade importante para divulgar artigos publicados anteriormente em *História, Ciências, Saúde – Manguinhos*. Por exemplo, nos 50 anos do Plano Nacional de Imunizações e do lançamento do Movimento Nacional pela Vacinação, por parte do Ministério da Saúde em 2023, divulgamos trabalhos da história da vacinação no Brasil e na América Latina. Também o dia mundial da alimentação (16 de outubro) atraiu muitas visitas para o dossiê temático sobre a história da alimentação lançado em 2021.

Nossas redes sociais mantiveram o ritmo de publicações de três a quatro textos por semana, a maioria de divulgação de novos artigos ou eventos de interesse do campo da história da ciência e de estudos de patrimônio. Um dos textos de grande repercussão foi a entrevista com a pesquisadora Maria Paula Meneses, do Centro de Estudos Sociais da Universidade de Coimbra, Portugal, que veio ao Rio de Janeiro participar do oitavo Congresso da Rede de Popularização da Ciência e da Tecnologia na América Latina e no Caribe (RedPop), ocorrido entre 10 e 16 de julho de 2023 e organizado pelo Museu da Vida (Casa de Oswaldo Cruz/Fiocruz). Cabe ressaltar que membros da equipe da revista – Marina Leme, Vivian Mannheimer e Roberta C. Cerqueira – participaram desse congresso com uma apresentação sobre os 10 anos dos *blogs* e das redes sociais de *História, Ciências, Saúde – Manguinhos*, completados em 2023.

Continuamos com o compromisso de publicar de acordo com as diretrizes da ciência aberta, o qual inclui mudanças no processo editorial, como a aceitação de originais previamente publicados em servidores de *preprints*. Infelizmente, a maioria dos historiadores, incluindo editores de periódicos, ainda considera que os *preprints* divulgam investigações inacabadas, colocam em xeque o ineditismo dos artigos, comprometem a avaliação imparcial dos originais e divulgam resultados de pesquisa em velocidade ainda alheia à disciplina. Argumentamos que a valorização do formato *preprint* deve ser promovida e incentivada por editores. Além de encurtar o tempo entre a pesquisa e sua comunicação pública, os *preprints* possuem o potencial de promover debates. Também fortalecem avaliações responsáveis e bem documentadas e registram a formulação, a seleção de dados e o amadurecimento das ideias das pesquisas científicas.

Outra novidade na forma de *História, Ciências, Saúde – Manguinhos* publicar seus textos diz respeito aos artigos com autoria de duas ou mais pessoas, que passam a informar as contribuições de cada uma delas seguindo critérios internacionais como conceituação, coleta de dados, interpretação e redação. A iniciativa procura garantir a ética na produção do texto científico e que este seja resultado efetivo de uma parceria, e não de uma relação de orientação (como infelizmente por vezes acontece). Estamos cientes de que os periódicos científicos de história ainda devem fazer mais progressos na ciência aberta e responder a desafios complexos, como promover os pareceres abertos e assegurar a diversidade e equidade nos artigos aprovados.

Um dos desafios de *História, Ciências, Saúde – Manguinhos* em 2024 é a conclusão do processo de migração para um novo sistema *online*, o Open Journal Systems (OJS). O sistema permite gerenciar a submissão, o fluxo de trabalho editorial e a publicação *online* dos artigos. Com um processo editorial inteiramente digital, o sistema permite a redução de gastos com impressão, garantindo a gratuidade aos autores que submetem artigos à revista.

Por fim, gostaríamos de ressaltar que os avanços da revista só foram possíveis graças à equipe editorial (Mônica Auler, Camilo Papi, Mônica Cruz Caminha, Vinícius Renaud, Marciel Mendonça, Miriam Junghans, Marina Lemle, Vivian Mannheimer e Fernando Vasconcelos). A equipe da revista fica muito grata aos autores, pareceristas, editores de seção e membros do conselho editorial que permitiram a nosso periódico ter especial visibilidade e prestígio, tanto no Brasil como em outros países da América Latina e de outras partes do mundo. Nosso especial reconhecimento a Marcos José de Araújo Pinheiro, diretor da Casa de Oswaldo Cruz, unidade da Fiocruz, onde trabalhamos, pelo valioso apoio à revista.
